# Broadband Anti-Reflective Coating Based on Plasmonic Nanocomposite

**DOI:** 10.3390/ma9080636

**Published:** 2016-07-28

**Authors:** Mehdi Keshavarz Hedayati, Moheb Abdelaziz, Christoph Etrich, Shahin Homaeigohar, Carsten Rockstuhl, Mady Elbahri

**Affiliations:** 1 Nanochemistry and Nanoengineering, Institute for Materials Science, Faculty of Engineering, Christian-Albrechts-Universität zu Kiel, Kiel 24143, Germany; moab@tf.uni-kiel.de (M.A.); shho@tf.uni-kiel.de (S.H.); 2 Institute of Condensed Matter Theory and Optics, Abbe Center of Photonics, Friedrich-Schiller-Universität Jena, Max-Wien-Platz 1, Jena 07743, Germany; Christoph.Etrich@uni-jena.de; 3 Institute of Theoretical Solid State Physics, Karlsruhe Institute of Technology, Wolfgang-Gaede-Str. 1, Karlsruhe 76131, Germany; carsten.rockstuhl@kit.edu; 4 Institute of Nanotechnology, Karlsruhe Institute of Technology, P.O. Box 3640, Karlsruhe 76021, Germany; 5 Nanochemistry and Nanoengineering, Helmholtz-Zentrum Geesthacht, Geesthacht 21500, Germany; 6 Nanochemistry and Nanoengineering, School of Chemical Technology, Aalto University, Kemistintie 1, Aalto 00076, Finland

**Keywords:** antireflective coating, plasmonic nanocomposite, absorbing antireflective coating, antireflection

## Abstract

We report on the fabrication, the characterization, and the optical simulation of a gold–silica nanocomposite and present its integration into a broadband anti-reflective coating (ARC) for a silicon substrate. The two-layer ARC consists of a nanocomposite (randomly distributed gold cluster in a silica matrix) and a pure silica film. We capitalize on the large refractive index of the composite to impose an abrupt phase change at the interface of the coating to diminish the light reflection from the substrate through the ultrathin nanocoating. The average reflectivity of the silicon can be reduced by such a coating to less than 0.1% in the entire visible spectrum. We experimentally and numerically prove that percolated nanocomposites with an overall thickness of 20 nm can provide anti-reflectivity up to near infrared (NIR). The ARC bandwidth can be shifted more than 500 nm and broadened to cover even the NIR wavelength by changing the volume filling fraction of the gold clusters. The angular sensitivity of thin ultrathin antireflective coating is negligible up to 60°. The present ARC could find applications in thermo-photovoltaics and bolometers.

## 1. Introduction

Reflection of light from the interface between two media is a property that is desirable in some optical devices such as mirrors, but it is mostly unfavorable. For example, in displays the surface reflection causes undesirable 'ghost images' [1] and in solar cells the reflection corresponds to efficiency loss [2,3], while the high reflectivity is desirable in advertising displays [4] and some LEDs [5]. The trend to develop coatings—which reduce the reflection at the interfaces, known as antireflective coating (ARC)—has been progressing for many years. In this context, in a classical theoretical proposal developed in 1879, Lord Rayleigh showed that the reflectivity from any surface can be lowered whenever the refractive index (RI) contrast between the media on adjacent sides of the interface is minimized [6].

Generally, the reflection of p- and s-polarized light at the planar interface between two semi-infinite, homogeneous, isotropic media is governed by the Fresnel coefficients [7]. For glare reduction, two common methods are texturing the surfaces [8,9,10] or providing a gradient RI layer atop of the substrate [11,12]. Recently, an absorbing coating has been presented as a new ARC, which shows a strong reflection reduction (due to absorption) at optical frequencies [13,14]. Although such an approach is not useful for optical devices such as glasses and telescopes, they can find application in energy harvesting devices [13,15] and surface reflectance coloring [16]. Plasmonic ARC is another emerging ARC, which has been proposed for solar application. There, the large metal particles act as scattering centers and thereby increase the light, which reaches the substrate [17,18,19,20,21].

We have recently demonstrated an ultrathin plasmonic ARC, where the reflection drop is caused by the interference of the waves within the layers or the absorbing nature of the coating [22] while the coating is much thinner than the conventional quarter or half-wavelength coatings [23]. The latter liberates designers and manufacturers from the traditional thickness constraints of ARC. In such a hybrid ARC, two geometries of classical ARC are combined with a plasmonic ARC to provide a very broadband anti-glare coating (for details see [24]). Eventually, the dispersive nature of the plasmonic coating in spectral proximity to the resonance frequency provides quasi-two different geometries at the wavelengths shorter and longer than the plasmonic resonance wavelength. Therefore, one could have two different arrangements of RI of the layers in one design. In previous studies, the applicability of a layer structure consisting of an Ag–SiO_2_ nanocomposite was shown. Here, we experimentally and numerically demonstrate the strong dispersive character of ultrathin plasmonic gold–silica nanocomposite coating with low imaginary part *k* and strongly dispersive real part *n* of the refractive index [23]. The high refractive index of the gold–silica composite enables us to shift the operational wavelengths of the ARC to the near infrared (NIR).

## 2. Results and Discussion

It is known that double layer dielectric film (e.g., SiO_2_/TiO_2_) ARC can reduce the reflectivity of optical materials such as glass, quartz [25], or silicon as long as the materials with lower refractive indexes face the air (to provide a gradual gradient in RI from the air to the substrate) [26]. However, we have proven recently that it is possible to perceive ARCs where the RI of the top layer is larger than that of the second layer [22].

In general, if the optical thickness of the layers in double-layer geometry obeys the following equations:

(1) $$n_{1}\times d_{1}=n_{2}\;\times \;d_{2}$$

The necessary and sufficient index condition in order to reduce reflection down to 0 is [27]:

(2) $$n_{1}\times n_{2}=n_{0}\times n_{s}$$

where *n*_0_, *n*_1_, *n*_2_, and *n*_s_ are the RIs of the air (environment), first (upper) layer, second layer, and substrate, respectively, and *d*_1_ and *d*_2_ represent the thickness of the first and second layer, respectively. In transparent (lossless) dielectric coating, the optical phase changes that causes interference is run by gradual growth inside the layers. The phase change at the interface is always 0 or π depending on the RI contrast between the media on adjacent sides of the interface. However, if the dielectric is replaced by an absorbing medium, e.g. a plasmonic composite, the phase change at the interface can be some value other than 0 or π, hence liberating the design from conventional thickness restriction. So, such non-trivial boundary phase shifts let the total phase buildup reach almost 0 for layers considerably thinner than λ/4*n*, forming an absorption resonance [14]. In other words, films (sub-wavelengths) thinner than a quarter-wavelength can possess antireflection properties [22].

**Figure 1:**
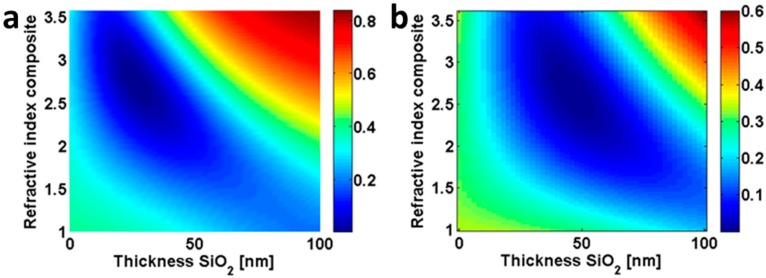
Reflection contour of a 20 nm film with various refractive indexes (RIs) on the SiO_2_ film with different thicknesses at a wavelength of (**a**) 500 nm and (**b**) 700 nm.

Figure 1 shows a calculated reflection contour of 20 nm film of varying RI versus the thickness of the second layer on top of a silicon substrate at two different wavelengths (500 nm (Figure 1a) and 700 nm (Figure 1b)). It is apparent that the minimum reflection (dark blue color) can be realized in what we call reverse-Rayleigh geometry, when the top layer has a higher RI than the second layer. Indeed, an asymmetric Fabry–Perot configuration is established when the lossless dielectric (SiO_2_) is sandwiched between silicon and TiO_2_. As outlined above, the high RI contrast of the top layer with air gives rise to a large phase shift of the directly reflected beam with respect to the light reflected at the following interface. Therefore, both contributions interfere destructively and cancel the reflection. Furthermore, the low RI of the second layer (for a suitably chosen thickness) provides the required optical path for the incident way to interfere constructively into the substrate. This enhances the light transmission into the substrate.

**Figure 2:**
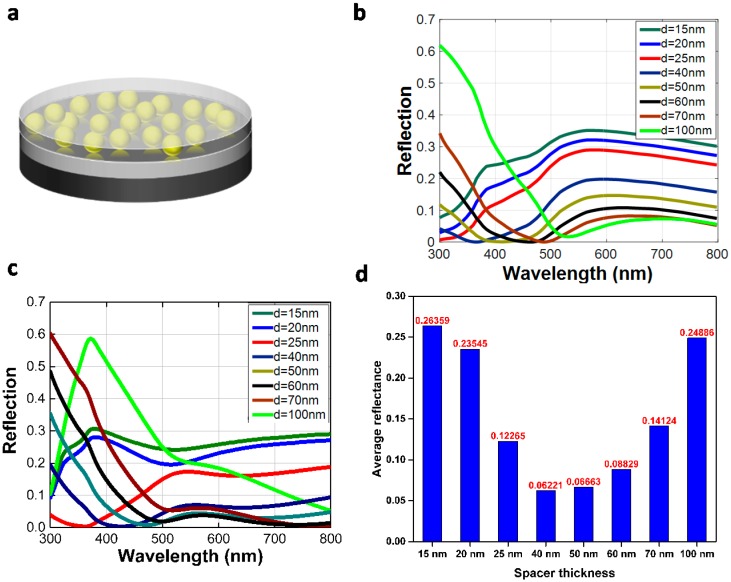
(**a**) A schematic geometry of the designed coating on a silicon wafer substrate; (**b**) Simulated and (**c**) experimental reflection spectra of 20 nm Au–SiO_2_ (40%) deposited on silicon in which the thickness of spacer layer is varied from 15 to 100 nm; (**d**) The average reflectance spectra of the data are shown in (**c**).

We suggested that designing a broad band ARC ultrathin coating requires a large RI and strong dispersive coating that is absent in natural materials, yet can be achieved by implementing plasmonic nanocomposite. Here, we consider the use of a plasmonic nanocomposite made from gold as an integrated part of a bi-layer antireflection structure on top of a silicon substrate. The schematic geometry of the plasmonic ARC used in this work is depicted in Figure 2a. It consists of two layer coatings. The top layer facing air is a 20 nm ultrathin nanocomposite (Au–SiO_2_), which is coated atop of a thin SiO_2_ film (second layer) with a silicon wafer as the substrate. The nanocomposite in which gold nanoparticles with 4–5 nm in diameter are encapsulated in a silica matrix are fabricated with co-sputtering (see the experimental procedure). The details of fabrication of such a nanocomposite, TEM preparation, and calculation of the effective properties are described in detail elsewhere [23,28].

Here, the effective properties of gold–silica nanocomposite with 40% filling fraction of gold were calculated. For this purpose, the finite-difference time-domain has been used to simulate the reflection and transmission from the thin layer of the actual nanocomposite. By inverting the complex reflection and transmission coefficients, effective properties for the nanocomposite could be retrieved [23]. This allows consideration of it in a subsequent design process of the coating. For the design of the coating, we have used a thin layer transfer matrix method (see Section 3) to simulate the optical properties of the multilayer system on top of a silicon substrate (Figure 2b). Figure 2c shows the experimental verification of the reflection spectra of Au–SiO_2_ nanocomposite deposited on silicon while the thickness of the spacer layer is varied. Relatively good agreement between the simulation (Figure 2b) and experiment (Figure 2c) are observed. Note that the agreement between simulation and experimental data does not apply for every detail. In particular, we notice a continuous red-tuning of the spectral features in the simulation (Figure 2b) when compared to the experimental results (Figure 2c). The most reasonable explanation we have for that at the moment is the overestimation of the dispersion in the effective properties of the composite material, i.e., the resonances are too strong. Here, the dispersion refers to the real part of the effective permittivity. This implies that longer wavelengths are sufficient to observe spectral features that emerge experimentally at shorter wavelengths. This is the largest difference we see between simulation and experiment. This overestimation of the resonance strength can be explained by the assumption of perfect spherical shape of the objects in the simulation to render an actual implementation of the composite. This does not withstand experimental reality. There, the composite is made from objects that are more dispersive and diverse in their actual geometry than assumed in the simulation. This broadens the resonances and actually weakens the resonance strength. This translates to less dispersive material properties, and this causes the appearance of spectral features in the experiments at lower wavelengths than are observed in the simulation at longer wavelengths.

In all the cases, the average reflectance through the measured wavelengths are relatively low and even went down to around 0.06 when the spacer thickness was 40 nm (Figure 2d). We attributed the origin of the anti-glare properties of the sub-wavelength nanocomposite to the strong dispersive nature of the coating. The wavelength at which gold particles show a plasmon resonance is located around 550 nm. At longer wavelengths, the RI of a composite containing these particles jumps abruptly despite its drop at shorter wavelength. Hence, above the resonance wavelength, the RI of the top layer is much higher than the second layer, and reverse-Rayleigh configuration is established [22]. In other words, two antireflection dips are formed below and above the plasmon resonance wavelength, as clearly shown in Figure 2b,c. Thus, by tuning the spacer layer thickness while keeping the filling fraction and the thickness of the plasmonic nanocomposite constant, we are able to tailor the ARC frequencies at visible and NIR frequencies.

**Figure 3:**
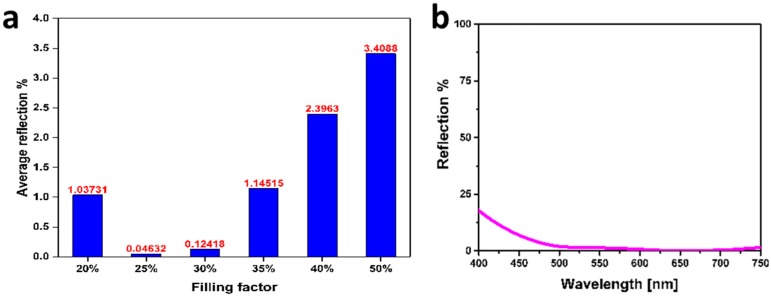
(**a**) Average reflection at visible wavelengths of 20 nm Au–SiO_2_ nanocomposite deposited on 70 nm SiO_2_ on a silicon substrate wherein the filling fraction is changed from 20% to 50% gold; (**b**) Reflection spectra of 20 nm Au–SiO2 (25%) on 70 nm SiO_2_ spacer layer in visible spectra.

**Figure 4:**
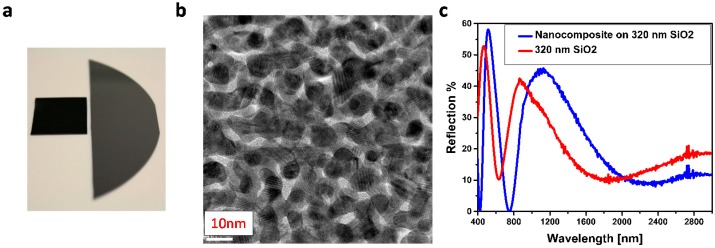
(**a**) True photograph of the anti-reflective coating (ARC) coated silicon (left) in comparison to a bare silicon wafer (right); (**b**) Top-view TEM image of 20 nm thick near percolating Au–SiO_2_ nanocomposite; (**c**) Reflection spectra of 20 nm Au–SiO_2_ composite, which is deposited on a 320 nm thick spacer layer (blue) in comparison to 320 nm SiO_2_ coated silicon.

Reduction of surface reflection using the hybrid concept requires precise control over the parameter of the layers with respect to thickness and filling fraction. For instance, tuning the filling fraction of the nanocomposite would change the reflection response as well as the ARC properties of the coating. Figure 3a illustrates the experimental reflection measurement (averaged over visible spectra) results of the plasmonic ARC as a function of filling fraction, while all other parameters are kept constant. In this context, it is obvious that the 25% filling fraction is the optimum concentration of gold in the nanocomposite for a spacer layer thicker than 70 nm SiO_2_ film (Figure 3b) which results in realization of a black silicon (Figure 4a).

Thus, a broadband antireflection occurs only under this optimum condition and any deviation from the optimum value increases reflection. For instance, if the filling fraction goes beyond 50% (see the TEM graph in Figure 4b) the composite turns to a semi-continuous metallic film and becomes highly reflective [29]. More specifically, there is an interplay between the photonic response of the overall film and the plasmonic response of the top layer. Above 30% filling fraction of gold nanoparticles, the plasmonic response (resonance reflection [30]) dominates and, accordingly, reflection increases instead of diminishes.

Figure 4c shows the reflection of silicon covered with 320 nm SiO_2_ in comparison with the same coating with an extra 20 nm percolated nanocomposite (40% filling fraction) as the top layer. The SiO_2_ coated sample shows the typical trend of an interference coating, where the reflection at the three wavelengths is reduced, however, not entirely suppressed. The deposition of the plasmonic nanocomposite atop the SiO_2_ film reduces the reflection and gives rise to photonic shift that gradually increases while going from visible to NIR. Indeed, one can see a significant shift of the reflection dips from 632 to 748 nm and 1864 to 2368 nm, which corresponds to 116 and 504 nm shift of the dip, respectively, upon deposition of ultrathin nanocomposite on silica. This gradual increase in the photonic shift is owing to tailoring the real part of the refractive index of the nanocomposite [11]. Thus, it is obvious that the plasmonic nanocomposites can act as a high *n* coating which allows tailoring the photonic response of the traditional ARC coating.

**Figure 5:**
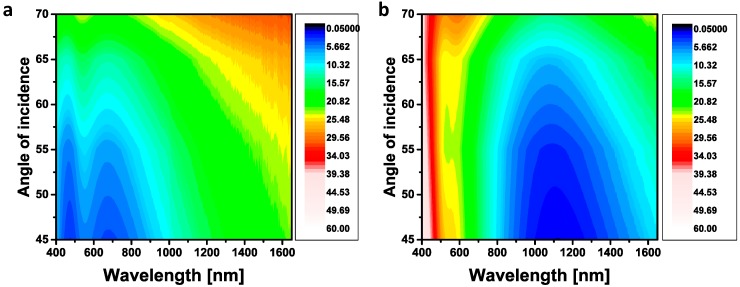
Reflection spectra of 20 nm Au–SiO_2_ (40%) deposited on (**a**) 60 nm and (**b**) 130 nm SiO_2_ on silicon substrate.

Iridescence is a critical problem for optical devices [11] and in particular in thermo-photovoltaics [31]. Our designed ARC is less sensitive to the angle of incidence. Since it is an ultrathin coating, the optical path of the light, even at glazing incidence, does not differ considerably to that of normal incidence. So, it is expected that such a sub-wavelength coating is almost insensitive to the angle of incidence at wavelengths where the reflection is low. Ellipsometric characterization [30,32] of the stack of percolated composite deposited on 60 nm (Figure 5a) and 130 nm (Figure 5b) SiO_2_ on silicon substrate at different angles of incidence also confirmed that the reflectivity remains low even at large angles up to 60°.

## 3. Materials and Methods

### 3.1. Fabrication of Nanocomposite

The ARC consists of a silicon wafer as base layer, spacer layer (SiO_2_), and nanocomposite layer (gold inclusion in a SiO_2_ matrix). Co-sputtering technique was used for sample preparation. This method allows deposition of nanocomposites with different filling fractions and thicknesses [28,33]. Inside the chamber, both magnetrons were arranged with an angle of 50° relative to the sample holder. In order to acquire a uniform thickness and homogeneous metal distribution, the sample holder kept spinning during co-deposition. An RF power supply was used for deposition of the polymer matrix and a DC power supply for metallic inclusions. To control thickness and filling fractions of films, both magnetron sources were calibrated carefully. The initial vacuum of each film was determined by doing some preliminary experiments. Accordingly, the depositions were done in a vacuum around 7.4 × 10^−5^ mbar and 1.6 × 10^−6^ mbar for spacer layer and nanocomposite layer, respectively.

### 3.2. Optical Characterization

Optical characterization of all the deposited samples were carried out by a UV-Vis-NIR spectrometer (Lambda900, PerkinElmer; Waltham, MA, USA). The reflectance measurements were done with the commercial compartment provided by the machine manufacturer, and aluminum mirror was used as references. In order to assess the absolute reflectance, the values were corrected upon normalizing the measured data to the reflectance values provided by the manufacturer. Ellipsometry analysis of the dielectric films was assessed with an automated angle M-2000^®^ Ellipsometer (J. A. Woollam Co., Lincoln, NE, USA). For modeling of the optical properties, the software provided by the company (CompleteEase, Lincoln, NE, USA) was used. Film thickness was measured by profilometer as well as ellipsometer.

### 3.3. Simulation

An ordinary thin film transfer-matrix method was used. It is described in many standard textbooks of optics [34]. At its heart, it considers the optical action of a thin film made from a homogenous, local, isotropic, and linear medium in terms of a 2 × 2 matrix. The entries of the matrix describe the evolution of the tangential field component through the film with a given thickness and made from a material with a given permittivity. Since these tangential field components are continuous at the interface, a stratified medium, i.e., a sequence of layers, is easily represented by the product of the individual 2 × 2 matrices characterizing each layer. Coupling to the layer stack of the substrate, where the field is only a plane wave corresponding to transmission, and the superstrate, where the field is a superposition of a forward propagating (incident field) and a backward propagating (reflected field) plane wave, allows derivation of two equations with two unknowns, that correspond to the reflected amplitude and the transmitted amplitude normalized to the amplitude of the illumination. From these amplitudes all further quantities can be derived. Permittivity and thickness of each layer in these layer stacks can be easily modified. This was done in the manuscript in order to obtain the quantitative insights documented in Figure 1.

## 4. Conclusions

In short, we have shown that the high dispersive nature of plasmonic nanocomposite consisting of gold nanoparticles in a silica matrix can perform as a visible wavelength ARC structure and it could reduce the glare to NIR wavelengths. The former originates from the plasmonic resonance of the coating while the latter is attributed to the photonic nature (high refractive index) of the coating. Hence, we suggest to use the percolated nanocomposite as a high *n* and low *k* (loss) material for broadening the application of traditional ARC to NIR wavelengths. While our finding would enhance our understanding for designing of low loss plasmonic medium, the concept that has been introduced here can be practically used for applications where reflection in a wideband of spectrum is unfavorable.
